# Consecutive Serious Medical Concomitants Associated With Extremely Severe Anorexia Nervosa in a Patient With Body Mass Index of 10.2 Kilograms per Square Meter: A Case Report

**DOI:** 10.7759/cureus.55749

**Published:** 2024-03-07

**Authors:** Takeshi Yamashita, Takahiko Fukuchi, Hitoshi Sugawara, Yoshiro Okajima, Masahiro Hiruta

**Affiliations:** 1 Department of Comprehensive Medicine 1, Saitama Medical Center, Jichi Medical University, Saitama, JPN; 2 Department of Diagnostic Pathology, Saitama Medical Center, Jichi Medical University, Saitama, JPN

**Keywords:** interdisciplinary approach, cognitive dysfunction, gelatinous marrow transformation, liver dysfunction, refractory hypoglycemia, feeding and eating disorders, anorexia nervosa (an)

## Abstract

Anorexia nervosa (AN) is often accompanied by numerous medical complications and mental disorders. There are few specialized AN facilities in Japan, resulting in the unmet medical needs of patients with AN. A 37-year-old Japanese woman was admitted to the hospital after experiencing a disturbance of consciousness. Her body mass index was 10.2 kg/m^2^. She developed the following serious medical concomitants associated with extremely severe AN: hypothermia, shock liver, refractory hypoglycemia, acute gastric mucosal bleeding, gelatinous marrow transformation, catheter-related bloodstream infection and infective endocarditis due to β-lactamase-negative* Staphylococcus aureus*, aspiration pneumonia, intracranial hemorrhage, candidemia, and osmotic demyelination syndrome in the pons, which led to a fatal condition that quickly worsened after we started treatment.

The patient was able to overcome several serious concomitants and be discharged from the hospital after multidisciplinary treatment team care. AN is associated with increased rates of all-cause mortality. It is important to take an interdisciplinary approach with emergency physicians, intensivists, hematologists, gastroenterologists, psychiatrists, clinical psychologists, a nutrition support team with a nationally registered nutritionist and hospitalists, and hospitalization as required based on appropriate medical evaluation with good patient and family rapport. Furthermore, social and educational efforts aimed at preventing the development of AN are necessary.

## Introduction

Anorexia nervosa (AN) is one of the feeding and eating disorders characterized by abnormally low body weight by restriction of energy intake relative to requirements, intense fear of gaining weight or of becoming fat, distorted perception of body weight or body shape, and persistent lack of recognition of the seriousness of low body weight resulting in a range of medical complications, including hypoglycemia and the refeeding syndrome [1]. All-cause mortality is 4 to 14 times greater in patients with AN compared to the general population, and medical complications of AN account for approximately 60% of deaths in patients with AN [2]. In a study that examined the mortality of anorexia nervosa in 201 people, the standardized mortality ratio for AN with a minimum body mass index (BMI) of 10.5 kg/m^2^ or less was reported to be 44.6 [3].

Japanese women with AN reported a comparable age of onset of clinically significant eating disorder, and when compared to the US data, the onset of eating pathology in Japan is approximately two years later [4]. AN can adversely affect almost every body system. Weight loss and malnutrition can both cause complications. Various complications associated with AN have been reported [5,6]. For instance, liver dysfunction frequently occurs at the nadir weight of patients. This generally represents apoptosis, a form of programmed hepatocyte death caused by malnutrition [5]. Hematological cytopenia occurs because of gelatinous marrow transformation (GMT), with atrophy of the normal fat content in the marrow and replacement with mucopolysaccharides [5]. Patients with life-threatening AN may be forcibly treated with total parenteral nutrition (TPN), including intravenous hyperalimentation may be associated with catheter-related bloodstream infection (CRBSI) [7]. In patients with AN, brain atrophy occurs due to malnutrition, and neurocognitive functioning may be permanently impaired even if brain atrophy improves with weight restoration [8]. Additionally, bradycardia, hypotension, postural hypotension, and hypothermia signify a compromise secondary to being underweight [6].

There have been several reports of individual complications caused by AN to date [5,6]. As far as we have searched the literature, there are no reports concerning serious consecutive concomitant medical disorders caused by AN. Finding an appropriate treatment facility is difficult because AN can cause fatal physical complications due to psychiatric manifestations and feeding and eating disorders.

We herein present the case of a patient with sequentially developed medical complications of extremely severe AN, including severe hypoglycemia, liver dysfunction, GMT, shock liver, gastrointestinal bleeding, intracranial hemorrhage due to thrombocytopenia, infective endocarditis, and candidemia from CRBSI. The patient was able to overcome these serious concomitants and be discharged from the hospital after multidisciplinary treatment team care.

## Case presentation

A 37-year-old Japanese woman was admitted to the hospital with a disturbance of consciousness. On the day of admission, she saw her daughters off to school at 7:30 a.m.; when they returned home at 3:00 p.m., they discovered the patient lying on her back in a room on the second floor. After waiting for her in-laws, the patient was transported to our hospital by ambulance.

The patient’s family history was unremarkable. The patient has no history of smoking or drinking alcohol. She was a registered dietitian who was unemployed. She lived with her husband and two daughters, aged 12 and 10 years. She had two pregnancies and two births. Her body weight was approximately 45 kg in her early 20s. Her body weight was less than 40 kg after giving birth to her second child at age 28, and she developed amenorrhea. When she was 33 years old, her BMI was 15.2 (weight 35.5 kg, height 153 cm). Her weight then continued to decrease to about 31 kg. Her husband said that when her weight reached 32 kg, she said that she had gained weight and was worried about her weight. In the month before her hospitalization, she lost around 8 kg on a low-calorie diet.

Her consciousness was impaired when she arrived at the emergency department at 4:15 p.m., and her Glasgow Coma Scale (GCS) score was E1V1M4. Her BMI was 10.2 kg/m^2^ (weight, 23.9 kg), bladder temperature was 24.6 °C, and her blood pressure (BP) was unmeasurable; however, the carotid arteries were palpable. Her saturation of percutaneous oxygen was unmeasurable. The patient’s heart rate was 48 bpm and regular. She did not breathe spontaneously. She was assisted respiration with a bag-valve-mask resuscitator and was intubated immediately.

A physical examination revealed yellowing of the ocular conjunctiva and poor skin turgor. There were no odontoparallaxis or finger calluses. The results of laboratory tests conducted upon admission are shown in Table 1. Her blood culture at the time of the emergency room was negative.

**Table 1:** Laboratory test results and body weight on admission, discharge and two years later HPF, high-power field; AST, aspartate aminotransferase; ALT, alanine aminotransferase; LD, lactate dehydrogenase; ALP, alkaline phosphatase; PT-INR, international normalised ratio of prothrombin time; APTT, activated partial thromboplastin time; n/a, not available.

	Admission		Discharge		Two years later	Reference range			Admission	Discharge	Two years later	Reference range
	Compete Blood Count								Biochemistry			
White blood cell	5.56		2.03		2.13	3.5–9.1 × 10^3^/µL	Total protein		5.7	6.2	6.5	6.6–8.1 g/dL
Neutrophils	76		58		61	40%–74%	Albumin		3.7	3.9	4.3	4.1–5.1 g/dL
Lymphocytes	22		34		29	19%–48%	Total bilirubin		1.69	0.65	0.57	0.4–1.5 mg/dL
Monocytes	1		6		9	3.4%–9%	Direct bilirubin		1.01	0.21	0.17	0.05–0.23 mg/dL
Eosinophils	0		0		0	0%–7%	AST		2,336	24	28	13–30 U/L
Red blood cell	405		393		379	376–500 × 10^4^/µL	ALT		1,364	17	21	7–23 U/L
Hemoglobin	13.3		11.9		12.4	11.3–15.2 g/dL	LD		1,344	352	262	124–222 U/L
Hematocrit	40.6		37.0		37.3	33.4%–44.9%	Creatine kinase		985	1377	189	41–153 U/L
Platelet	9.2		23.3		19.6	13–36.9 × 10^4^/µL	ALP		492	398	123	106–322 U/L
	Urinalysis and Sediments						C-reactive protein		0.02	0.02	0.04	0.00–0.14 mg/dL
Gravity	1.014		1.011		n/a	1.005–1.025	Urea nitrogen		46	9	19	8-20 mg/dL
pH	7.0		5.5		n/a	5–7.5	Creatinine		0.59	0.40	0.51	0.46–0.79 mg/dL
Protein	1+		-		n/a	Negative	Sodium		142	140	140	138–145 mmol/L
Red blood cells	1-4/HPF		n/a		n/a	Negative	Potassium		4.5	4.0	4.2	3.6–4.8 mmol/L
White blood cells	1-4/HPF		n/a		n/a	Negative	Chloride		102	107	105	100–110 mmol/L
	Coagulation tests						Corrected calcium		8.2	8.9	8.6	8.4-10.1 mg/dL
PT-INR	1.63		n/a		n/a	0.9–1.2	Inorganic phosphorus		7.7	5.1	3.6	2.7-4.6 mg/dL
APTT	67.4		n/a		n/a	28.5–40.9 second	Magnesium		3.3	1.8	1.9	1.7-2.5 mg/dL
							Plasma glucose		12	79	86	70–109 mg/dL
Body weight	23.9		35.3		35.0	kg						
Body Mass Index	10.2		15.3		14.9	kg/m^2^						

Her ventilator settings in the intensive care unit (ICU) were mode, assist-control ventilation; tidal volume, 350 ml; respiratory rate, 20 breaths/min; the fraction of inspired oxygen (FiO2), 0.3; and positive end-expiratory pressure (PEEP), 5 mmHg. After a 50% glucose infusion from a central venous (CV), the random plasma glucose (PG) level increased from 12 mg/dL to 106 mg/dL. The patient was given large volumes of balanced crystalloid fluid. Her systolic BP, measured with a placed arterial line, was maintained at approximately 100 mmHg by noradrenaline infusion. Noradrenaline was administered at an initial dose of 8 mg/h and was tapered off over eight hours after the mean arterial pressure stabilized above 65 mmHg.

**Figure 1:**
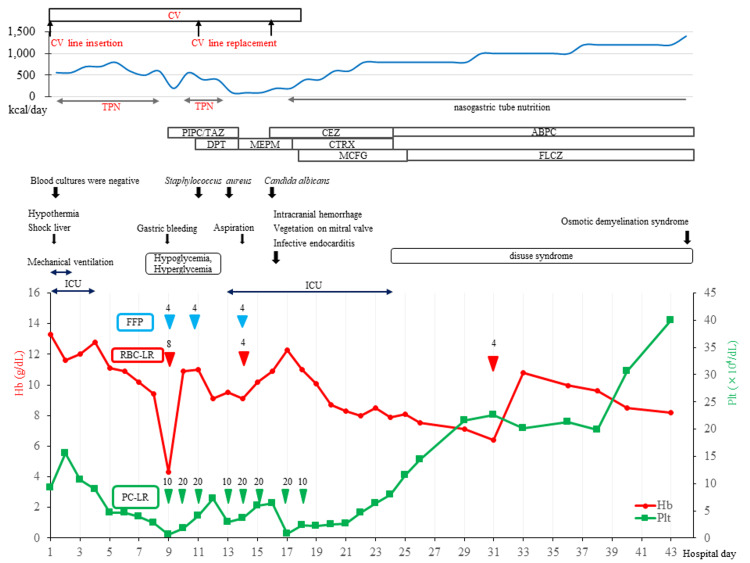
The clinical course The clinical course of the progress of periods of central venous (CV) insertion, caloric intake including total parenteral nutrition (TPN) and nasogastric tube nutrition, antibiotic treatments, intensive care unit (ICU), transfusions for cytopenia, and the levels of hemoglobin (Hb) and platelet count (Plt). Antibiotics: Piperacillin/tazobactam (PIPC/TAZ), daptomycin (DPT), cefazolin (CEZ), meropenem (MEPM), ceftriaxone (CTRX), micafungin (MCFG), ampicillin (AMPC), and fluconazole (FLCZ). Transfusion: Red Cells Concentrate, Leukocytes Reduced (RBC-LR), Platelet Concentrate, Leukocytes Reduced (PC-LR), and fresh frozen plasma (FFP).

Figure 1 shows the patient’s clinical course. The day after admission, the patient’s consciousness improved, and her GCS score was E3E5V6; she was extubated. Her body temperature had recovered to 36°C. Systolic BP was maintained at > 100 mmHg without noradrenalin infusion. Her liver function gradually improved to aspartate aminotransferase (AST), 1,383 U/L; alanine aminotransferase (ALT), 1,245 U/L; and lactate dehydrogenase (LD), 1,042 U/L; however, the patient continued to have hypoglycemia.

She was administered TPN through a CV catheter. On hospital day 1, 560 kcal of ELNEOPA-NF No.1 Injection 1,000 ml (120 g of glucose, 20 g of total free amino acids, electrolytes, comprehensive vitamins, and trace element solutions for high-calorie infusions) was administered over 24 hours. TPN was administered to her during CV catheter insertion but was discontinued after CV catheter removal.

From hospital day 3, she was offered a diet of 750 kcal/day, of which she could eat 50 to 80%, resulting in an oral energy intake of 375 to 600 kcal/day. To prevent refeeding syndrome, blood tests were performed daily to correct hypophosphatemia, hypokalemia, and hypoglycemia. Oral caloric intake was gradually increased. Her random PG levels remained between 100 and 200 mg/dL until the hospital day 8.

On hospital day 4 after leaving the first ICU stay, she was able to tell us her previous eating habits. She was afraid of becoming obese. She wondered what to feed her children and occasionally ate too much. The patient reported no auto-induced vomiting. She exhibited abnormal eating behavior and a distorted body shape perception, but had never visited a clinic and had never been diagnosed with AN. These findings met the diagnostic criteria for extremely severe AN per the Diagnostic and Statistical Manual of Mental Disorders 5th Edition [1] and the International Classification of Diseases 11th Revision [9]. We diagnosed her with AN with the help of a psychiatric specialist. We cared for her and her family from hospital day 4 with psychiatrists and clinical psychologists and made nutritional adjustments with nutritionists.

**Figure 2:**
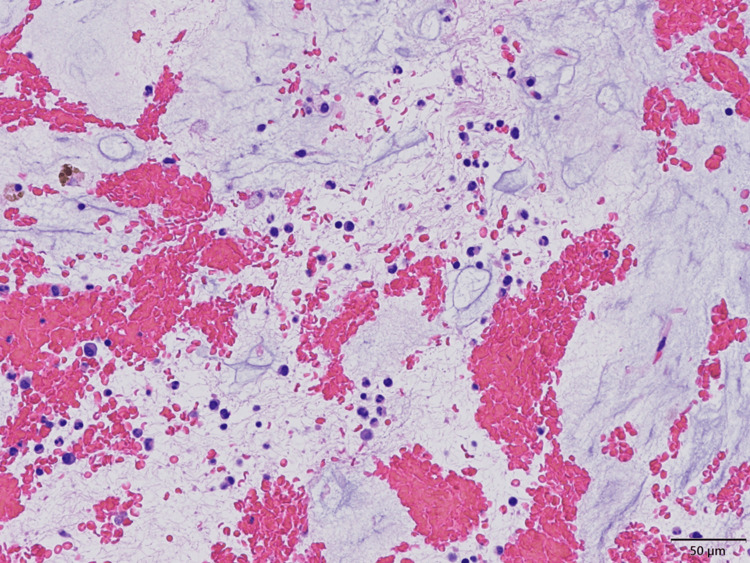
Pathological findings of bone marrow aspiration Pathological findings of bone marrow aspiration stained with Hematoxylin and Eosin (original magnification, 200X) revealed significant gelatinous marrow transformation (GMT); however, megakaryocytes were difficult to identify and no hematopoietic colonies were observed.

On hospital day 9, her hemoglobin (Hb) level dropped from 9.4 g/dL to 3.5 g/dL, and her platelet count gradually decreased to 6,000/µL. The patient was administered transfusions of red blood cells leukocytes reduced, platelet concentrate leukocytes reduced, and fresh frozen plasma. The same day, her random PG level fluctuated widely from 40 mg/dL to 555 mg/dL. She continued to have hypoglycemia of around 50 mg/dL and was given frequent glucose replacement. On hospital day 10, upper gastrointestinal endoscopy revealed acute gastric mucosal bleeding in several locations at the center of the gastric fundus; however, there were no ulcers or erosions. Bone marrow aspiration revealed marked GMT; however, megakaryocytes were difficult to identify, and no hematopoietic colonies were observed (Figure 2). Transfusions were needed to treat anemia and thrombocytopenia.

On the night of hospital day 10, the patient had a fever of 38℃. On hospital day 11, blood cultures were obtained and piperacillin/tazobactam (PIPC/TAZ) 18 g/day was administered. On the following day, blood cultures showed cluster-forming gram-positive cocci and daptomycin 150 mg/day was added to PIPC/TAZ. Finally, on hospital day 13, we detected β-lactamase-negative *Staphylococcus aureus*, i.e. both methicillin-susceptible and penicillin-susceptible *Staphylococcus aureus*, and treated the CRBSI with a de-escalation to 6 g/day of cefazolin.

**Figure 3:**
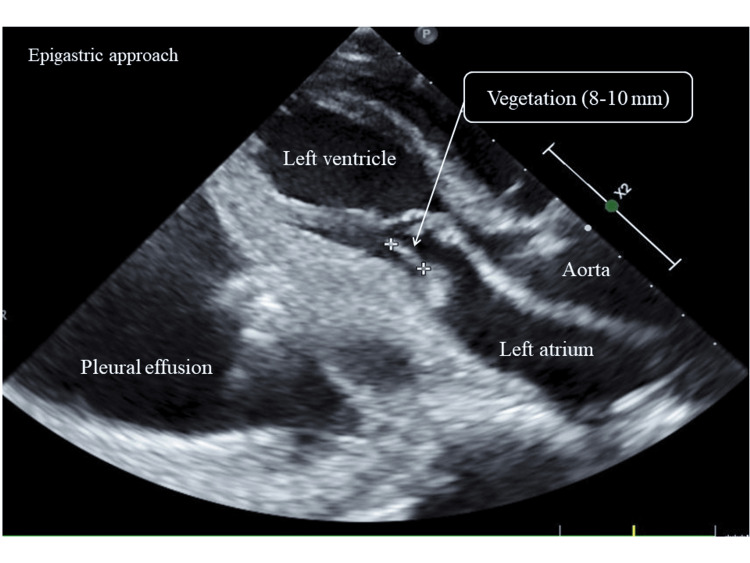
Transthoracic echocardiography on hospital day 16 Transthoracic echocardiography on hospital day 16. This showed vegetation measuring 8–10 mm on the posterior apex of the mitral valve.

On hospital day 14, the patient developed aspiration pneumonia due to sputum blockage caused by dysphagia in the AN [10]. Sputum was aspirated by bronchoscopy and she was treated with high-flow oxygen. As the patient’s condition worsened on PIPC/TAZ, we switched to meropenem (MEPM) 3 g/day for the treatment of hospital-acquired pneumonia. Blood cultures performed on hospital day 13 were again found positive for *Staphylococcus aureus* on hospital day 15. On hospital day 16, transthoracic echocardiography (TTE) revealed vegetation measuring 8-10 mm on the posterior apex of the mitral valve (Figure 3).

**Figure 4:**
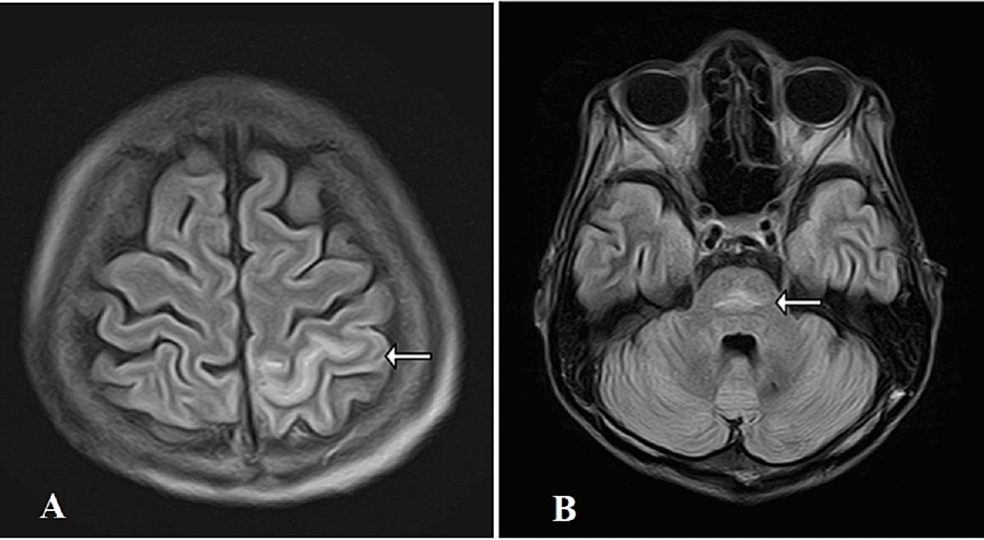
FLAIR MRI of the head On hospitalization day 14, head magnetic resonance imaging (MRI) in a fluid-attenuated inversion recovery (FLAIR) sequence showed a high signal area in the left parietal lobe (Figure 4A). On hospitalization day 44, head MRI on FLAIR showed high signal area in the pons (Figure 4B).

Her physical examination revealed no presence of Osler’s node, splinter hemorrhage, or mucosal bleeding. Two blood cultures taken more than 12 hours apart were positive for *Staphylococcus aureus*, which together with the echocardiographic findings of the vegetation on the mitral valve led to the diagnosis of infective endocarditis after meeting the modified Duke criteria for a definite case. Head magnetic resonance imaging (MRI) was performed in search of cerebral embolism, which is a frequent complication. Fluid-attenuated inversion recovery (FLAIR) head MRI (Figure 4) showed a high signal area in the left parietal lobe (Figure 4A) consistent with cerebral hemorrhage. We considered that the intracranial hemorrhage was caused by a bleeding tendency based on the imaging findings and clinical courses, such as coagulation abnormality and thrombocytopenia. MEPM was changed to ceftriaxone 4 g/day, considering its transferability into the cerebrospinal fluid.

The result of the blood culture performed on hospital day 16, reported on hospital day 18, did not show *Staphylococcus aureus*. However, a yeast-like fungus was detected. We administered micafungin 150 mg/day to the patient. Finally, on hospital day 22, the culture revealed *Candida albicans*, and the patient did not have endophthalmitis related to candidemia. We treated her infections of *Staphylococcus aureus* and *Candida albicans* with ampicillin 12 g/day and fluconazole 400 mg/day because of antibiotics de-escalation for 42 days after the blood cultures became negative.

On hospital day 17, nasogastric tube nutrition for her was started at 200 kcal, and the dose was increased by 200 kcal every two days. On hospital day 23, after nasogastric tube nutrition reached 800 kcal, the dose was increased by 200 kcal every week.

On hospital day 24 after leaving the second ICU stay, we noticed that the patient was unable to bend her bilateral elbows and knees. This was thought to be disuse syndrome associated with prolonged bed rest. Psychotherapy and counselling by a psychiatrist and a clinical psychologist have been provided to her since the time she left the ICU. Psychiatrist, clinical psychologist, ward nurses, and hospitalists continued to listen to her and her family's wishes as best they could, although she had difficulty communicating with them.

On hospital day 44, a brain MRI was performed to determine the difficulty of improving disuse syndrome. The parietal hemorrhage was found to be absorbed; however, FLAIR revealed a high-signal area in the pons (Figure 4b). Based on the MRI findings, we determined that osmotic demyelination syndrome (ODS) was possible [11].

From hospital day 44, the dose of nasogastric tube nutrition was 1,400 kcal. On hospital day 55, oral intake from jelly was started in parallel with nasogastric tube nutrition. She did not refuse to eat by mouth. The nutritionist confirmed her preferences and changed the dietary formula. On hospital day 60, the patient was started on a 1,200 kcal diet and was able to eat all of her food. Nasogastric tube nutrition was reduced along with the amount of oral food intake and was discontinued on hospital day 73.

The brain MRI on hospital day 65 showed improvement with the disappearance of the high-signal areas in the pons. On hospital day 75, TTE revealed no vegetation. Oral intake was increased to 1,400 kcal on hospital day 80, and 1,800 kcal was fully eaten on hospital day 83. On hospital day 87, we assessed her cognitive function for the first time using the Hasegawa Dementia Scale-Revised (HDS-R) (30-point scale) and the Mini-Mental State Examination (MMSE) (30-point scale) [12]. A perfect score on the HDS-R and MMSE is 30, and a normal adult takes a perfect score. The HDS-R score was reduced to 24, within the normal range, and the MMSE score was reduced to 24, classified as mild cognitive impairment. Her score was deducted due to recent memory impairment.

Starting on hospital day 123, the patient received 2,000 kcal and continued to eat the full amount until discharge. These dietary patterns and calorie content were coordinated by the nutrition support team (NST) with national registered nutritionist. Frequent blood tests were performed on her to check her electrolytes and replenish them as needed, and she did not develop refeeding syndrome.

On hospital day 129, HDS-R score improved to 29 and the MMSE score improved to 28 after long-term physical, occupational, and speech-language rehabilitation. Her neurocognitive function likely declined due to AN, because cognitive performance improves after weight gain in children and adolescents with AN [13]. After hospital day 151, the patient was transferred to another hospital for physical therapy rehabilitation to prepare for returning home and discharged 10 days later. Her weight increased from 23.9 kg on admission to 35.3 kg at the time of discharge, and her BMI increased from 10.2 kg/m^2^ on admission to 15.1 kg/m^2^ at the time of discharge. The complete blood count at discharge was as follows: WBC,2030 × 103/µL; Hb, 11.9 g/dL; and platelet, 23.3 ×104 /µL.

The patient visited a mental health outpatient clinic once a month and was able to maintain a body weight of 35 kg without any eating disorder. Two years after discharge, the laboratory findings were as follows (Table 1): WBC, 2.130 × 103/µL; Hb, 12.4 g/dL; platelet count, 19.6 ×104 /µL; random PG, 86 mg/dL; total protein, 6.5 g/dL; albumin, 4.3 g/dL; AST, 28 U/L; ALT, 21 U/L; and LD, 262 U/L.

## Discussion

This patient developed consecutive serious medical concomitants associated with AN, namely hypothermia, refractory hypoglycemia, shock liver, acute gastric mucosal bleeding, gelatinous marrow transformation, catheter-related bloodstream infection due to β-lactamase-negative *Staphylococcus aureus*, aspiration pneumonia, intracranial hemorrhage, infective endocarditis caused by β-lactamase-negative *Staphylococcus aureus*, candidemia, and osmotic demyelination syndrome in the pons, which led to a fatal condition quickly becoming progressively worse since we start the initial treatment.

**Figure 5:**
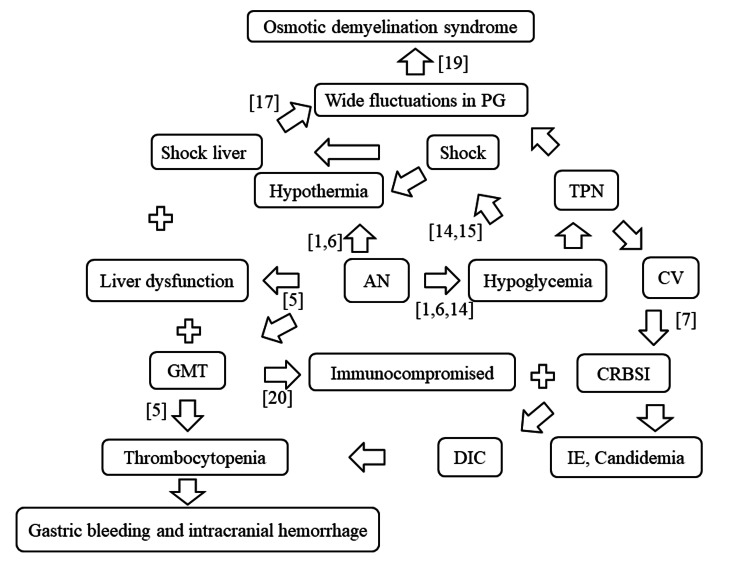
The proposed pathophysiology in the patient with AN The proposed pathophysiology, especially liver function, gelatinous marrow transformation (GMT), and hypoglycemia, directly affected multiple organ failure of anorexia nervosa (AN) in this patient. Abbreviations: central venous (CV), plasma glucose (PG), total parenteral nutrition (TPN), catheter-related bloodstream infection (CRBSI), disseminated intravascular coagulation (DIC), and infective endocarditis (IE).

However, she was able to successfully recover and gain weight with antibiotic treatment and an interdisciplinary approach. Interdisciplinary treatment team care is important [5]. Her general condition was managed by emergency physicians and intensivists during the acute/critical phases, and by hospitalists during general hospitalization. Hematologists were responsible for hematologic and bone marrow evaluation, and gastroenterologists for gastrointestinal endoscopic evaluation. Psychiatrists and clinical psychologists provided mental health care for the patient and her family, and the hospitalists and nurses on the unit talked with her to establish a good relationship. Physical rehabilitation was performed with physical, occupational, and speech therapists. Dietary formula and calorie content were coordinated by NST with national registered nutritionist. All of this was organized by the hospitalists and resulted in her surviving, getting discharged, and living at home. Figure 5 shows the proposed pathophysiology, particularly liver function, GMT, and hypoglycemia, which directly affected multiple organ failure in this patient with AN.

Hypoglycemia due to AN causes impaired consciousness [14]. Generally, bradycardia is observed in patients and reflects a heightened vagal tone in the setting of significant weight loss [15]. Hypothermia is another symptom of AN, and several researchers have recently challenged this view, suggesting that hypothermia and hyperactivity are central to AN [16]. The patient had bradycardia and hypovolemic shock, which resulted in shock liver and severe hypothermia. AN is often associated with liver dysfunction [5], which may worsen owing to shock. Acute liver injury, such as shock liver, is associated with excessively prolonged PT, thrombocytopenia, and an extremely high risk of hemorrhage [17]. In AN, bone marrow examination revealed bone marrow hypoplasia and GMT [5]. This was combined with disseminated intravascular coagulation due to CRBSI, resulting in severe thrombocytopenia. Thrombocytopenia occurred due to intracranial hemorrhage, gastric bleeding, and severe anemia. The possibility that hypothermia, hypoglycemia, and shock liver are not directly caused by AN cannot be ruled out. However, it is unlikely that the hypothermia, hypoglycemia, and shock liver were caused by a bloodstream infection complicated by septic shock. Because blood cultures were negative on admission.

The symptoms of liver failure include fluctuating PG levels due to difficulty in storing glycogen [17]. After admission, the patient continued to have low PG levels because of liver failure and AN. High PG levels are caused by a high-calorie infusion used for nutritional management in the treatment of hypoglycemia. ODS is an uncommon neurological disorder caused by damage to the myelin sheath of brain cells [18]. ODS was reportedly caused by malnutrition, chronic liver disease, hypoglycemia, and hyperglycemia [18]. As the patient had kept an appropriate serum sodium level, we hypothesized that the ODS, in this case, was caused by significant fluctuations in PG [19]. The report shows no improvement in cognitive function in AN in adulthood [13]. However, her cognitive function improved over the course of her hospitalization. It is possible that the improvement may have been due to a combination of factors because of the improvement in her general condition, nutritional status, and ODS.

AN causes lymphocytopenia and decreased lymphocyte activity, resulting in immunocompromised immunity [20]. AN becomes severe when an infection occurs [20]. The patient underwent CV catheter insertion for systemic management. For AN, TPN, including CV catheter associated with CRBSI [7]. CRBSI may occur because of the CV catheter, liver failure, and malnutrition. The immunocompromised patient with AN and the replaced CV catheter further developed infective endocarditis and candidemia. AN has a high mortality rate, and cardiac disease is a risk factor for death [5]. In this case, all symptoms were treated appropriately, and the patient was able to improve without cardiac disease.

## Conclusions

AN is associated with increased rates of all-cause mortality. Interdisciplinary treatment team care and hospitalization of patient with AN, including appropriate medical evaluation for AN with good patient and family relationship, is important to overcome the serious multiple medical concomitants associated with AN. Furthermore, social and educational efforts aimed at preventing the development of AN are necessary.
